# Modelling the climatic niche of turtles: a deep-time perspective

**DOI:** 10.1098/rspb.2016.1408

**Published:** 2016-09-28

**Authors:** Amy M. Waterson, Daniela N. Schmidt, Paul J. Valdes, Patricia A. Holroyd, David B. Nicholson, Alexander Farnsworth, Paul M. Barrett

**Affiliations:** 1 School of Earth Sciences, University of Bristol, Wills Memorial Building, Queens Road, Bristol BS8 1RJ, UK; 2 School of Geographical Sciences, University of Bristol, University Road, Bristol BS8 1SS, UK; 3 Museum of Paleontology, University of California, 1101 Valley Life Science Building, Berkeley, CA 94720, USA; 4 Department of Earth Sciences, The Natural History Museum, Cromwell Road, London SW7 5BD, UK

**Keywords:** testudine, ecological niche model, niche stability, Late Cretaceous

## Abstract

Ectotherms have close physiological ties with the thermal environment; consequently, the impact of future climate change on their biogeographic distributions is of major interest. Here, we use the modern and deep-time fossil record of testudines (turtles, tortoises, and terrapins) to provide the first test of climate on the niche limits of both extant and extinct (Late Cretaceous, Maastrichtian) taxa. Ecological niche models are used to assess niche overlap in model projections for key testudine ecotypes and families. An ordination framework is applied to quantify metrics of niche change (stability, expansion, and unfilling) between the Maastrichtian and present day. Results indicate that niche stability over evolutionary timescales varies between testudine clades. Groups that originated in the Early Cretaceous show climatic niche stability, whereas those diversifying towards the end of the Cretaceous display larger niche expansion towards the modern. Temperature is the dominant driver of modern and past distributions, whereas precipitation is important for freshwater turtle ranges. Our findings demonstrate that testudines were able to occupy warmer climates than present day in the geological record. However, the projected rate and magnitude of future environmental change, in concert with other conservation threats, presents challenges for acclimation or adaptation.

## 1. Introduction

Global climate change over the last century has altered rainfall patterns and produced warming not observed for millennia. Future scenarios predict an increase in global temperature, relative to the pre-industrial era that will likely exceed 1.5–2°C by 2100 [1]. Climate plays a major role in determining biogeographic distributions [2,3] and, consequently, recent global environmental change has caused geographical range shifts in numerous species. However, large differences in the capacity of organisms to adapt or acclimatize have been recognized [4]. The impact of climate is expected to be especially important for terrestrial and freshwater ectotherms, such as amphibians and reptiles, whose body temperatures are tightly linked to their external environment [5,6]. The potential impacts of climate change on these animals have received considerable attention, and the ability to cope with local shifts in temperature and precipitation is expected to vary between taxa [7–9].

Forecasting future responses to climate change is challenging. The fossil record offers long-term distributional, ecological, and species-richness data that provide critical information for elucidating the effects of changing climate on palaeobiogeographic patterns, thereby informing our understanding of future ecological response [10–12]. A fundamental assumption of using the fossil record for this purpose is that the climatic niche occupied by an organism has remained stable through space and time [13]. However, the assumption of climatic niche stability has frequently been questioned given the long-term potential for evolutionary adaptation [14]. Ordination techniques and ecological niche models (ENMs) can be used to quantify climatic niches and niche stability by relating species occurrence records with climate variables [15,16]. These methods have been used to assess niche dynamics over quaternary and neogene timescales [17–19], but application to the deep-time fossil record remains limited [20,21]. In this study, we use the term niche to refer to the multivariate space of climate variables that best corresponds to observed taxon distributions and the associated distribution of potential abiotically suitable habitats [22].

Turtles, tortoises, and terrapins (collectively termed testudines) originated in the Late Triassic (approx. 220 Ma) [23] and have persisted through a wide range of changing climates. Of 335 recognized modern species, 40% are regarded as globally threatened or endangered [24]. Alongside overexploitation and habitat loss, climate change is a significant threat to their conservation status with ontogenetic growth rates, species abundance, and geographical ranges all predicted to decline under future climate scenarios [25,26]. Temperature places strong physiological constraints on testudine activity patterns and regulates sex determination of offspring [27]. Freshwater species distributions are closely linked with precipitation owing to their dependence on standing water availability [5,28]. Understanding how future environmental change may affect the group is therefore crucial for informing adaptive conservation management strategies.

The Late Cretaceous (approx. 72.1–66.0 Ma) record of testudines provides us a model system for testing possible testudine response to a future warmer climate. This interval was warmer than present, with a reduced equator-to-pole temperature gradient owing to higher polar temperatures, fewer seasonal extremes, and greater precipitation [29]. Well-calibrated global climate model simulations and a broad distribution of climate proxy data for model validation provide a good understanding of palaeoclimatic conditions [30,31]. The fossil record of testudines has a wide geographical coverage that exceeds modern-day distributions. The largest number of fossils can be found in the last two million years of the Maastrichtian (approx. 72.1–66.0 Ma) [32–34]. To test for the impact of climate on testudine niche limits, we use ENMs to estimate the modern and Maastrichtian niches and calculate the overlap between model projections. We identify non-analogous climates and apply an ordination framework to quantify metrics of niche unfilling (niche space occurring only in the Maastrichtian), expansion (niche space occurring only in the modern day), stability (niche space occurring in both time periods), and test for niche equivalency (*sensu* [16]). Our study focuses on two extant testudine freshwater families with pre-Maastrichtian origins (Trionychidae and Chelydridae) and two ecotypes freshwater, representing a wide range of families and terrestrial, representing Testudinidae and the extinct Nanhsiungchelyidae (electronic supplementary material, table S6), rather than identifying species-level ecological traits. We use these families and ecotypes as proxies of the species and the roles that they fulfil within an ecosystem and how the availability of potential suitable climate space changes, or remains stable, for these over time. This study provides the first application of ENMs and ordination methods to quantify the climatic niche dynamics of any Mesozoic vertebrate group. The application of these techniques to deep-time distributions provides baseline data for testudine niche limits in a past greenhouse that can inform our understanding of long-term ectotherm biogeographic response under fundamentally different climatic regimes to today, both in the past and under future scenarios.

## 2. Methods

### (a) Testudine occurrence data

Modern testudine occurrence data were taken from the World Turtle Database [35]. Fossil occurrence data were downloaded from the Palaeobiology Database (PBDB; paleodb.org) through the Fossilworks portal (fossilworks.org) on 3 September 2015, using the search terms Testudinata and Cretaceous. The fossil data (major contributors—[36]) were restricted by removal of ichnotaxa (trace fossils), ootaxa (fossil eggs), and marine taxa, then subsetted to include only those occurrences falling between 66 and 72.1 Ma, leaving a dataset of Maastrichtian non-marine turtles comprising 877 taxonomic occurrences (743 identified at least to family level) in 321 PBDB collections (electronic supplementary material, dataset S1). Owing to limitations in the number of fossils identified to genera or species, we based our analyses on family-level identifications (referred to herein as taxonomic) and ecotype level (fully terrestrial or freshwater). This allowed for a comparison of family-specific niche traits with those of more generalist ecological groups. The fossil record represents a complex mixture of preservational and sampling biases (e.g. temporal variation in sedimentary rock volume, accessibility, and sampling effort) [37]. Biases inherent in the Maastrichtian testudine fossil record result in a set of available fossil occurrences that likely underestimate past global distributions and thus potential climate niche space. However, the Maastrichtian is well sampled in comparison with other stages of the Cretaceous and even younger parts of the geological record. The geographical spread of Maastrichtian testudine occurrences covers all the continents except Australia and Antarctica (electronic supplementary material, dataset S1) and matches that of other tetrapod clades except for the apparently genuine absence of turtles at high latitudes [33]. Moreover, the turtle carapace is highly durable and more likely to be preserved than the majority of other vertebrate skeletal elements [33,38].

The use of higher taxa as a surrogate for species data has been subject to debate [39], though family or higher-level distributional data have been used to effectively describe the environmental tolerances of some modern taxa [40,41]. Use of family data to investigate evolutionary processes in the fossil record is common as it increases the number, and geographical coverage of occurrence data [42,43]. The ecophysiological responses of Trionychidae and Chelydridae species to contemporary climate are broadly conserved at the family level and species within these families occupy similar habitats [44]. Trionychidae and Chelydridae are therefore considered a good surrogate for their associated species-level traits.

### (b) Climate data

Modern climate variables were derived from UKMO unified model HadCM3; a fully coupled atmosphere–ocean general circulation model. The model has a surface resolution of approximately 417 × 278 km at the equator, reducing to 295 × 278 km at 45° latitude. A detailed description of the General Circulation Model (GCM) can be found within [45,46]. Maastrichtian climate variables were derived from UKMO unified model HadCM3 L; identical to HadCM3 except for reduced spatial resolution in the ocean component [31]. A bilinear interpolation was applied to convert GCM climate variables to 10 min resolution. The GCM shows good broad agreement with proxy data, however, in continental interiors proxy-model comparisons consistently report conditions that are too extreme, i.e. cold winters and hot summers [47,48]. Thus, interpretation of niche analyses within these regions should be treated with caution. Climate variables were chosen to reflect abiotic niche characteristics that have been shown to be ecologically limiting to modern testudine distributions [26,28] and that can be feasibly determined in the fossil record. Initially, a larger number of temperature and precipitation-related variables were considered (electronic supplementary material, table S1). However, to reduce collinearity between variables [49], we retained the combinations with a Pearson's pairwise correlation coefficient of less than |0.7|. Three variables were used in the final niche analyses: mean temperature of the coldest quarter, and precipitation of the wettest and driest months.

### (c) Niche modelling and quantifying niche change

The MaxEnt ENM algorithm was used to model modern and Maastrichtian testudine climatic niches as it is well suited for use with presence-only occurrence data. Specifically, it compares the climatic conditions at locations of occurrence records with randomly selected points from a background extent to create maps of habitat suitability [50]. A fivefold cross validation procedure [51] was used to create global models for the modern and calculate area under the curve (AUC) statistics (predictive performance measure). AUC values of 1 indicate a perfect model prediction and 0.5 represents a no-better-than-random prediction [52]. Jackknife tests and % variable contribution were used to estimate variable importance. Modern ENMs were projected onto Maastrichtian climate layers. A binomial test was used to assess the ability of modern ENMs to predict Maastrichtian fossil occurrences. The same procedure was repeated to produce Maastrichtian ENMs and project these to modern climate layers. We used the Boyce index (BI), with values ranging from −1 to 1, to assess the ability of Maastrichtian ENMs to predict modern occurrences [53]. Geographical niche overlap was calculated between modern and Maastrichtian predictions [54] using both forward projection (FP) models to the modern and backward projection (BP) to the Maastrichtian. Geographical niche overlap ranges from 0 (no overlap) to 1 (identical predictions). Sensitivity analyses were performed to assess the impact of training extent on MaxEnt ENMs (electronic supplementary material, figures S12, S13, and table S7), and calibrations with modern species-level occurrence data for Trionychidae and Chelydridae were compared with family-level analyses (electronic supplementary material, figures S6–S11).

Non-analogue climates occur over time owing to climate change and result in unreliable projections of ENMs. We identify the presence of non-analogue climates using the ExDet software package that measures the similarity of variables between time slices by accounting for deviation from the mean and correlation between variables [55]. Measures of niche margin dynamics (unfilling, expansion, and stability) and environmental niche overlap between the time slices were quantified using an ordination framework that compares the environmental attributes at sites of organism occurrence, in different locations or at different time periods [16]. The framework uses a principal component analysis (PCA); niche quantification analyses are performed within the first two PCA axes [56]. We used a pooled-range approach [16] where analyses are made relative to the entire climate niche occupied in both time periods. We also used this framework to test for statistical significance of niche similarity and equivalency. Niche similarity assesses whether occupied climatic niches in two time periods are more similar than would be expected by chance and niche equivalency tests for whether climatic niches in two time periods are effectively identical [16]. These analyses were conducted in environmental space and provide a comparison with ENM analyses in geographical space. All ENM and ordination analyses were performed in the R statistics package (version 3.2.1), using the dismo and ecospat packages [57].

## 3. Results

### (a) Drivers of testudine climatic niches today and in the Maastrichtian

Mean AUC values for test data in the modern and Maastrichtian cross-validation ENMs were moderate to high (modern; Trionychidae: 0.83, Chelydridae: 0.92, terrestrial: 0.79, freshwater: 0.69 and Maastrichtian; Trionychidae: 0.94, Chelydridae: 0.98, terrestrial: 0.91, freshwater: 0.87) indicating that all models successfully discriminated the presence of the organisms from background locations. As expected, AUC values were lower for ecotypes compared with the taxonomic ENMs, as ecotypes include multiple testudine families, including some with more generalist ecological preferences. Temperature of the coldest quarter was the most important variable to modern ENM fit for Trionychidae (46.8%), freshwater (61.8%), and terrestrial (74.6%) ecotypes, based on MaxEnt mean % variable contribution and jackknife estimates. The explanative power of temperature was greatest for the terrestrial ecotype; this is expected as the presence of standing water (and thus precipitation) is less critical for terrestrial testudines primarily exploiting land environments. The precipitation-related variables showed significant importance for the freshwater ecotype (37.2%) and taxonomic ENMs (Trionychidae = 53.2%, Chelydridae = 83.4%), with precipitation of the driest month the most important variable overall for Chelydridae (61.9%). Temperature of the coldest quarter was the most important variable to Maastrichtian model fit for all testudine ENMs (Trionychidae = 80.2%, Chelydridae = 66.9%, freshwater = 65.2%, terrestrial = 60.9%). The precipitation-related variables combined also showed significant explanative power for both ecotype and taxonomic ENMs (terrestrial = 39.0%, freshwater = 34.9%, Chelydridae = 33.1%, Trionychidae = 19.7%) (electronic supplementary material, figure S2).

Sensitivity analyses highlight that ENM training extent has a significant impact on modern ENM projections (electronic supplementary material, figures S12 and S13). Modern ENMs with a global training extent better predicted modern testudine occurrences (electronic supplementary material, tables S2 and S7), therefore, results herein focus on these. Modelled modern environmental suitability at the family level is driven largely by the widest ranging species in Trionychidae and Chelydridae (electronic supplementary material, figures S6–S11). This suggests that using family-level occurrence data to predict suitable climate space in the past will likely identify the widest range of potential abiotically suitable habitats for these taxonomic groups.

### (b) Testing niche stability

**Figure 1.**
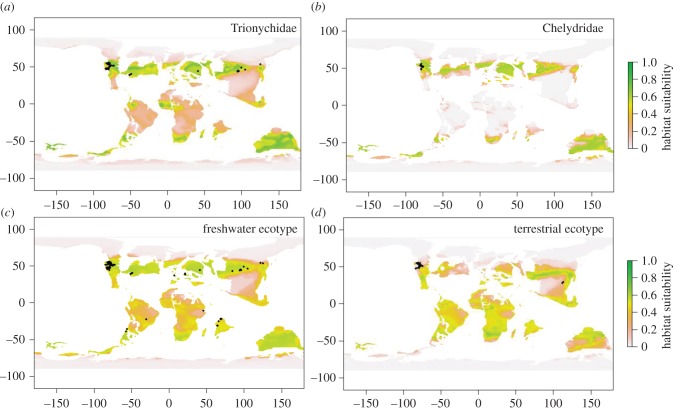
Modern MaxEnt ENMs projected to Maastrichtian climate layers. Maastrichtian testudine fossil occurrences (filled circles) overlay the maps of habitat suitability for Trionychidae (*a*), Chelydridae (*b*), freshwater ecotype (*c*), and terrestrial ecotype (*d*). Geographically filtered fossil occurrences (one per climate grid cell) are: Trionychidae: 60, Chelydridae: 19, freshwater ecotype: 111, terrestrial ecotype: 27. The continents are in a palaeoconfiguration, using a reconstruction following the methodologies in [31].

Modern ENMs were projected to Maastrichtian climate layers (BP models) and Maastrichtian ENMs to modern climate layers (FP models), to test for stability in climate niches between the two time periods. Modern ENM projections were statistically significant for the freshwater ecotype and taxonomic ENMs (binomial test results, *p* < 0.01 at all three omission thresholds: electronic supplementary material, table S2), suggesting stability in the climatic drivers of the niche between the modern and the Maastrichtian for these groups (figure 1). In contrast, a non-significant result for the terrestrial ecotype at the 50% omission threshold suggests this climatic niche is less stable (*p* = 0.09). Maastrichtian ENM projections (electronic supplementary material, figure S2) showed poorer predictive performance than modern ENMs, which may be a result of fewer occurrences in the Maastrichtian compared with the modern for ENM calibrations. Model predictive power may also be influenced by taxonomic differences in environmental tolerances (i.e. species versus family level; electronic supplementary material, figures S6–S11). Average Boyce indices were all negative (electronic supplementary material, table S3), suggesting model predictions of modern testudine occurrence data are no better than random. Chelydridae (BI: −0.24) and terrestrial ecotype (BI: −0.37) scores are most negative and indicate predictions of low suitability where presence is more frequent. BP models generally showed higher geographical niche overlap (defined between 0 and 1, with 1 being identical) than the FP models. Freshwater ecotype models showed the highest mean geographical niche overlap (BP: 0.66, FP: 0.65), followed by Trionychidae (BP: 0.51, FP: 0.44), Chelydridae (BP: 0.50, FP: 0.32), and terrestrial (BP: 0.40, FP: 0.54). Environmental niche overlap was 0.36 for Trionychidae, 0.30 for Chelydridae, 0.26 for the freshwater ecotype, and 0.17 for the terrestrial ecotype. Environmental niche overlap scores are expected to be lower than geographical niche overlap scores, because geographical niche overlap compares how ENM predictions of suitable areas overlap on the landscape, whereas environmental niche overlap quantifies observed overlap of occurrences in environmental space, thus not including those areas that are suitable but unoccupied.

**Figure 2.**
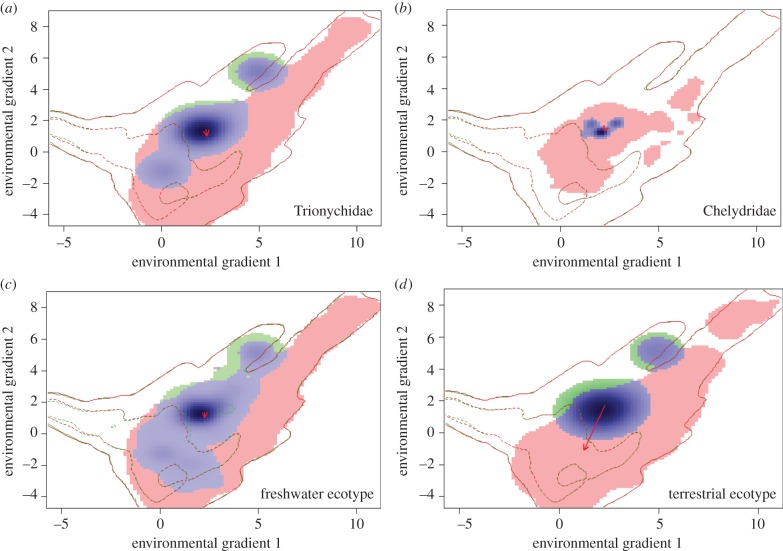
Testudine niche change between the Maastrichtian and the modern. Niche change in climate space is presented for Trionychidae (*a*), Chelydridae (*b*), freshwater ecotype (*c*), and terrestrial ecotype (*d*). Environmental gradient 1 represents 52.88% total variation and environmental gradient 2 represents 22.8% (contribution of original climate variables are shown in electronic supplementary material, figure S15). Solid contour lines illustrate the full range (100%) of climate space in the two time slices and dashed lines are 50%. A pooled-range approach was used [16], thus contour lines for the Maastrichtian (red) and modern (green) are identical. Shading shows the density of modern species occurrences per grid cell and the red arrow indicates the change in direction of the niche centre from the Maastrichtian to the modern. Blue pixels show niche stability (climate conditions occupied in both time periods), red pixels show niche expansion (climate conditions occupied in the modern only), and green pixels show niche unfilling (climate conditions occupied in the Maastrichtian only).

Change in niche margins within environmental space varies between the testudine groups (figure 2). The freshwater ecotype displays high stability (90%) and minimal expansion (10%). Trionychidae not only shows stability (67%), but also shows significant expansion (approx. 31%). In contrast, expansion is higher for Chelydridae (72%), with some stability (28%). The terrestrial ecotype shows the greatest expansion (77%) and lowest stability (23%). Measures of niche unfilling were low or non-existent for all groups except for the terrestrial ecotype (12%). Tests for niche similarity were statistically significant for Trionychidae, Chelydridae, and the freshwater ecotype but not statistically significant for the terrestrial ecotype (i.e. the hypothesis that the Maastrichtian and modern niches are no more similar than by chance cannot be rejected; electronic supplementary material, table S5). Tests for niche equivalency showed observed environmental overlaps were lower than 95% of simulated overlaps, rejecting the hypothesis of niche equivalency for all testudine groups. Overall, this shows that, with the exception of the terrestrial ecotype, the testudine groups occupy environmental niches that are similar but not identical between the modern and Maastrichtian (electronic supplementary material, table S5 and figure S14). Change of the terrestrial ecotype niche centre was primarily along environmental gradient 2, (figure 2 and electronic supplementary material, figure S15), suggesting that shifts in both temperature and precipitation influence the climatic niche change of terrestrial testudines from the Maastrichtian to the modern.

**Figure 3.**
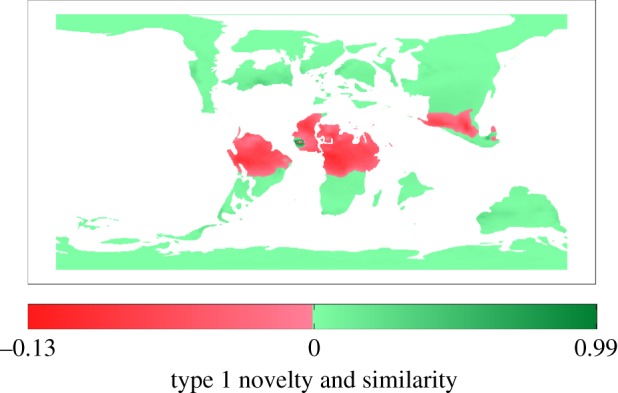
Availability of non-analogue climate space between the modern and Maastrichtian. Green, regions of similar climate variables (0 indicates maximum similarity), red, regions with at least one variable outside of the univariate range (type 1 novelty). The more negative values the type 1 novelty are, the less similar the climates are in these regions. (Online version in colour.)

Grid cells with at least one variable outside of the univariate range are confined to the low latitudes of South America, Africa, India, and southern Asia (figure 3). Temperature of the coldest quarter is most influential to type 1 novelty (electronic supplementary material, figure S3). Very few Maastrichtian testudine fossil occurrences fall within the non-analogue regions (five freshwater ecotype occurrences; electronic supplementary material, dataset S1); therefore, the influence of these areas on the model is expected to be minimal, and these regions were not excluded from our niche analyses. Environmental and occurrence data for the Maastrichtian represent a longer time period than does the modern data. This time-averaging increases the likelihood of sampling a greater proportion of the potential niche space. However, our analyses show that Maastrichtian turtles occur predominantly within the areas of suitable climate space, thus this does not appear to have affected our dataset.

## 4. Discussion

### (a) The role of temperature and precipitation in testudine climatic niches

Temperature explained most of the modelled habitat suitability in modern and Maastrichtian ENMs, confirming that thermal limits are the primary constraints on climate niches at the global scale [26,58]. Temperature links directly to testudine thermal physiology and is expected to place fundamental restrictions on distribution, as lethal temperatures exceed those suitable for reproduction [59]. We used temperature of the coldest quarter, thus ENM results suggest a more equable climate system during the Late Cretaceous meant that even high-latitude temperatures were greater than testudine critical thermal minima. The relative importance of temperature varies for different testudine groups as has been previously suggested [60]. Aquatic turtles appear to have lower and broader thermal limits than their terrestrial counterparts; minimum voluntary temperatures are estimated to be approximately 15°C for terrestrial testudinoids (tortoises), whereas some freshwater turtles can remain seasonally active in water as low as 5°C [59]. These physiological observations are supported by the greater importance of temperature in modern terrestrial ecotype ENMs compared with the freshwater groups. Precipitation-related variables were important in freshwater testudine ENMs, highlighting the key role of rainfall for turtles occupying aquatic ecosystems, which use water as a thermal buffer [27,61]. Today, the importance of precipitation is lowest for the terrestrial ecotype, supporting prior findings that terrestrial testudinoids show links with precipitation, but to a lesser extent than freshwater turtles owing to their terrestrialized life histories [58,62]. The explanatory power of temperature is greater in Maastrichtian ENMs compared with the modern, and precipitation shows significant contributions to Maastrichtian model fit in the ecotype ENMs. Whereas this could be a preservational bias resulting from fewer testudine occurrences in the Maastrichtian compared with the modern, it is important to note that land environments were wetter in the Maastrichtian than in the present day [29,32]. Therefore, the relative importance of total precipitation may not have been as large during a warmer and less seasonal global climate regime. Our results support the long-standing assumption that the occurrence of reptilian ectotherms indicate warm climate conditions [13,63]. However, precipitation is also critical in constraining the distributions of taxa that occupy freshwater environments.

### (b) Do climatic niches change over evolutionary timescales?

Few studies have addressed vertebrate ectotherm niche stability over evolutionary timescales [60,64]. Our results suggest climatic niche stability over deep-time varies among testudine groups. The freshwater ecotype and Trionychidae show the highest overlap in forward and backward ENM projections and significant niche margin stability between the Maastrichtian and the modern, implying that similar ecological limits have applied to these taxa for more than 70 million years. While the terrestrial ecotype and Chelydridae also displays niche margin stability, greater expansion suggests that the niche margins have shifted in environmental space between the two time periods for these groups (figure 2). Although the time interval sampled in the Cretaceous is longer than that sampled for the modern day, and thus should have greater potential for sampling niche spaces not represented in the modern day, Maastrichtian niches are smaller than those in the present. However, this could result from fewer sampled testudine occurrences in the Maastrichtian, which may represent a smaller range of suitable climate space in comparison with the modern testudine data. Niche expansion from the Maastrichtian to today for freshwater testudines is driven primarily by precipitation variables (figure 2*a*–*c*). Warmer temperatures and greater evaporation generated a more enhanced hydrological cycle in the Maastrichtian with continents that were approximately 15% wetter than present day [30]. At the regional scale, however, some areas (southwest North America, central South America, central and southern Africa, and central and southeast Asia) show higher wettest month rainfall today. These regions have high testudine species richness at present [65] and suggest that changing precipitation patterns may have played a key role in driving the expansion of freshwater turtle niche space between the Late Cretaceous and the modern day. Terrestrial ecotype niche expansion is in the direction of environmental gradient 2 (figure 2*d*), further indicating that as well as precipitation, thermal limits are important constraints on terrestrial testudines and likely influenced niche change from the Maastrichtian to the modern. Poor predictive performance of the terrestrial ecotype forward and backward ENM projections suggests that climate drivers of the modern and Maastrichtian niche differ significantly and/or that the climatic tolerances of terrestrial testudines may have undergone greater evolutionary change than in their aquatic counterparts. Adaptation to shifting aquatic environments during the Late Cretaceous could have played a key role in the success of freshwater testudines compared with terrestrial relatives that occupied more homogeneous habitats during this interval [34]. While observations of niche expansion may relate to evolution or adaptation in response to shifting abiotic conditions, it is important to acknowledge that changes in biotic interactions and accessibility to suitable habitats can also result in niche instability over time [22].

The retention of vertebrate ectotherm climate niche traits has primarily been tested over much shorter geological timescales, such as the most recent glacial [60]. For example, species-richness patterns of European reptiles and amphibians suggest niche stability between the Last Glacial Maximum and present day [66] and phylogenetic niche conservatism has been shown to influence the community structure of emydid turtle lineages in eastern North America [64]. Owing to the complex nature of climatic niches it has been suggested that some climate variables are subject to greater niche conservatism than others [67,68]. Araújo *et al*. [69] showed that tolerance to temperature maxima is largely conserved across lineages, whereas temperature minima varies between and within species. This is due to the definitive physiological limits posed by high temperatures, whereas the evolution of cold temperature tolerance may be more frequent and implies that ectotherm ranges are likely to be more sensitive to climate cooling rather than warming [7,70]. Niche stability results observed in some testudine groups support these findings and indicate that warmer stable climates may not necessarily be deleterious for testudines, in particular terrestrial testudinoids that display the highest thermal optima of the clade [59]. However, the ability of ectotherms to track the current rate of climate change via large-scale migration remains controversial [71].

### (c) Phylogenetic perspectives on niche change

The differences in niche stability between testudine groups appear to be under phylogenetic control. Trionychia is the most ancient cryptodire lineage (originating more than 145 Ma), but this clade did not dominate northern continental species assemblages until the Late Cretaceous [72]. Trionychidae originated in the Late Albian (approx. 113.0–100.5 Ma) and by the Maastrichtian its distribution had likely expanded to occupy a significant range of suitable climates. By contrast, Americhelydia (the larger clade including Chelydridae) underwent a rapid radiation during the Late Cretaceous, with the oldest chelydrid fossil recorded in the Albian (approx. 113–100.5 Ma) or Cenomanian (100.5–93.9 Ma) [73] but with clade diversification and geographical expansion occurring mainly in the Neogene [74]. More recently, evolving families may have expanded into climate spaces that differ from their Cretaceous relatives or ecotypes as they underwent diversification [75] and could account for lower climate niche stability for Chelydridae. Modern terrestrial testudinoids diversified after the K Pg boundary (66.0 Ma), during pronounced Palaeogene warm periods (Late Palaeocene thermal maximum and the Early Eocene climatic optimum) [76]. Maastrichtian terrestrial ecotype occurrences are of the herbivorous, tortoise-like Nanhsiungchelyidae, a sister clade to the Trionychidae that is not closely related to living Testudinidae; low environmental and geographical niche overlap (and non-significant niche similarity), in this group, strongly suggests that these early tortoise-like nanhsiungchelyids had different climate tolerances from living terrestrial turtles and from contemporaneous Cretaceous taxa that were predominantly omnivorous or carnivorous [34]. Consequently, variables other than temperature and precipitation may also have been important for delimiting geographical distributions (i.e. biome type and gross primary productivity) and could be tested using palaeovegetation models.

The climate niche stability observed in some testudine groups has implications for the use of turtles as palaeotemperature proxies in validation studies of GCM simulations of high-latitude warmth in the geological record [13,58]. While it suggests that long-established families that range through from the Mesozoic to today are indeed highly useful for such comparisons, caution should be applied when using fossils that represent testudine lineages originating or diversifying close to the palaeoclimate interval of focus.

The use of an ENM and ordination framework has enabled us to quantify modern and Maastrichtian testudine niches, and test for niche stability in a fundamentally different global climate to present day, providing novel insights into the group's biogeographic responses under climatic conditions that were warmer and wetter than modern. These methods have excellent potential for application to other vertebrate groups with fossil records exhibiting high preservation potential and good geographical coverage, enabling comparisons between the niche dynamics of other ectotherm and endotherm taxa, with those of extant relatives, over extended geological timescales. Here, we applied one type of GCM as this was the only appropriate climate model at the time of analysis, and we encourage future comparisons between multiple ENMs that use multiple GCM outputs (i.e. an ensemble approach), as these new GCMs become available for the Cretaceous. It is important to reiterate that our findings assess long-term niche dynamics occurring over lengthy timescales and hundreds of generations. Rates of climate change over the next century are projected to be more rapid and show greater magnitudes than has been experienced over millions of years of evolutionary history [1]. In concert with neo-ecological studies, our findings provide an important long-term perspective on testudine niche occupancy and climate. Given the current uncertainty regarding ectotherm migration or dispersal capacity and their long generation times [7], the potential for testudines to acclimatize or adapt undoubtedly poses significant future challenges for conservation management strategies.

## 5. Conclusion

Our results indicate that the retention of climate niche characteristics over evolutionary timescales varies among testudines. Temperature is the primary driver of modelled modern and Maastrichtian distributions at the global scale, but precipitation-related variables also play a significant role in delimiting freshwater turtle ranges. Niche change between the Maastrichtian and modern appears to reflect turtle phylogenetic relationships; longer-established groups show greater climate niche stability, whereas groups evolving closer to the Late Cretaceous display niche expansion and highlight that diversification within the clade likely led to the occupation of novel climate space.

## Supplementary Material

Supporting Information 1 (SI 1)

## Supplementary Material

Maastrichtian_turtle_PBDBdata.csv
