# Supplementary material for: Modelling the climatic niche of turtles: a deep-time perspective
Source: Proc Biol Sci. 2016 Sep 28;283(1839):20161408. doi: 10.1098/rspb.2016.1408 (PMC5046900; doi:10.1098/rspb.2016.1408)
Supplement: Supporting Information 1 (SI 1) [file rspb20161408supp1.docx]

Modelling the climatic niche of turtles: a deep-time perspective

Amy M. Waterson^1,2^, Daniela N. Schmidt^1^, Paul J. Valdes^2^, Patricia A. Holroyd^3^, David B. Nicholson^4^, Alexander Farnsworth^2^ & Paul M. Barrett^4^

**Supplementary Information**

**SI 1**

**Methods**

**Turtle occurrence data**

To allow for consistent comparison between the Maastrichtian testudine fossil occurrence data and GCM simulation we back rotated the modern day location for each testudine occurrence into their equivalent palaeo-position in the Maastrichtian. The rotations are based on the GETECH Plc. plate model and methodologies of Markwick and Valdes (2004); Markwick (2007). A recent study by Lunt, et al. (2016) also used this palaeorotation method for certain modern day proxy sample sites for comparison with model data.

**GCM climate data (HadCM3L)**

GCM data from HadCM3L were used to generate the climate variables for the Maastrichtian niche analyses. This simulation represents the “typical or “average” climate of the Maastrichtian (~72.1 – 66.0 Ma). The model is provided with key boundary conditions (paleogeography, solar constant, orbit and greenhouse gases) prior to running the simulation. The Maastrichtian GCM simulation in this study uses a palaeogeography that corresponds nominally to 69 Ma MP, but uses data for the whole Maastrichtian to develop GCM boundary conditions. A constant CO_2_ level is used throughout the GCM simulation (560 ppmv), the orbital configuration corresponds to present day and the solar constant is slightly lower (0.6%) than modern. To validate or test GCM output, comparison with proxy data is conducted following a completed model simulation. A description of the climate and comparison proxy data for a similar simulation can be found in ref. 39 (Hunter et al. 2013).

**Maxent ENM analyses**

We used MaxEnt to conduct ENM analyses; a five-fold cross-validation procedure was used (Elith et al. 2010) with default model parameters, with the exception of the regularization parameter which was set to 2.0 to avoid overfitting.

Maxent ENMs for modern projections to the Maastrichtian (and vice-versa) model calibrations for modern projections to the Maastrichtian (and vice-versa) used a global training extent. The geographical area used for model calibration is typically tied to specific hypotheses of the dispersal capacity of taxa being studied (Barve et al. 2011). Models trained using large study regions are likely to include areas of suitable conditions that are unoccupied due to biotic interactions and/or dispersal limitations, and therefore ENMs trained using broader extents have a tendency to over fit to conditions found near to occupied localities (Anderson & Raza, 2010). Or in other words, the larger the geographic area used for model training, the greater the number of environmental combinations that may be sampled as MaxEnt “background data”, and deemed “unsuitable”, limiting ENM predictions to a strict representation of the occupied niche. Where the goal is estimate the potential climatic niche, restricting calibration extent to a smaller region, based on the dispersal capacity of taxa, and then projecting this globally would typically be recommended. However in this case we found that a global calibration area performed best for our testudine groups (see sensitivity analyses below) and for this reason we calibrated the remainder of our ENM analyses with a global extent.

**Sensitivity analyses**

**Training extent**: we conducted sensitivity analyses using regional training regions in the modern (North America, Asia and Africa) and projected these to global modern climate layers for Trionychidae and the terrestrial ecotype (SI 1 Figures 8, 9 & Table 7). These results highlight that training region extent does have a significant impact on ENM projection to modern environmental layers, but in this case global modern calibrations (Figure 1) were able to better estimate global modern testudine distributions (SI 1 Table 2).

**Species-level vs family-level ENMs**: sensitivity analyses (SI 1 Figures 4-7) highlighted that there is some minor variation in the relative contribution of the environmental variables to species ENMs compared to family ENMs for Trionychidae and Chelydridae. Analyses highlight that modelled environmental suitability at the family-level is driven largely by the widest ranging species in each family, suggesting that using family level data to predict potential suitable climate space in the past is a reasonable approach, as this will mean we identify the widest range of potential suitable habitat for these taxonomic groups. It is also possible that the widest-ranging species are generalist turtle species, i.e. those with the largest geographic ranges, and that these species are most responsible for family-level ENM parameters. If this is the case, it is advantageous for our application to the fossil record as we would expect widespread generalists to be able to better occupy novel climate space and habitats (and also to have the richest and potentially best-sampled fossil records).

**Table 1** Pair-wise correlation heat map between considered climate variables; the upper diagonal cells show the correlation between modern covariates and the lower diagonal cells are the correlation coefficients for the Maastrichtian. bio1 = annual mean temperature, bio4 = temperature seasonality (max – min month), bio8 = mean temperature of wettest quarter, bio9 = mean temperature of driest quarter, bio10 = mean temperature of warmest quarter, bio11 = mean temperature of coldest quarter, bio12 = annual precipitation, bio13 =precipitation of the wettest month, bio14 = precipitation of driest month, bio15 = precipitation seasonality (coefficient of variation), bio16 = precipitation of wettest quarter, bio17 = precipitation of driest quarter, bio18 = precipitation of warmest quarter, bio19 = precipitation of coldest quarter.

| **ENM calibration** | **Average test AUC** | **Average binomial test** (projection to Maastrichtian): zero omission | **Average binomial test** (projection to Maastrichtian): 10% omission | **Average binomial test** (projection to Maastrichtian): 50% omission |
| --- | --- | --- | --- | --- |
| Trionychidae modern | 0.83 | p = < 0.01 | p = < 0.01 | p = < 0.01 |
| Chelydridae modern | 0.92 | p = < 0.01 | p = < 0.01 | p = < 0.01 |
| Freshwater modern | 0.69 | p = < 0.01 | p = < 0.01 | p = < 0.01 |
| Terrestrial modern | 0.79 | p = < 0.01 | p = < 0.01 | p = 0.09 |

**Table 2** Average test AUC scores for testudine MaxEnt modern ENMs, and average binomial test scores for projections of modern ENMs to Maastrichtian environmental layers, using zero, 10% and 50% omission thresholds.

| **ENM calibration** | **Average test AUC** | **Average Boyce index (projection to modern)** |
| --- | --- | --- |
| Trionychidae Maastrichtian | 0.94 | -0.07 |
| Chelydridae Maastrichtian | 0.98 | -0.24 |
| Freshwater Maastrichtian | 0.87 | -0.10 |
| Terrestrial Maastrichtian | 0.91 | -0.37 |

**Table 3** Average test AUC scores for testudine MaxEnt Maastrichtian ENMs and average Boyce indices for projections of modern ENMs to Maastrichtian environmental layers.

| **Turtle family/ecotype** | **Average geographic niche overlap – backward predictions (BP)** (Warren et al. 2009) | **Average geographic niche overlap – forward predictions (FP)** (Warren et al. 2009) | **Environmental niche overlap** (Broennimann et al. 2012) |
| --- | --- | --- | --- |
| Trionychidae | 0.51 | 0.44 | 0.36 |
| Chelydridae | 0.50 | 0.30 | 0.30 |
| Freshwater | 0.66 | 0.65 | 0.26 |
| Terrestrial | 0.40 | 0.54 | 0.17 |

**Table 4** Average geographic niche overlap for backward projection (BP) and forward projection (FP) Maxent ENMs, describing potential overlap in suitable geographic space for testudine families and ecotypes between the modern and Maastrichtian. Environmental niche overlap shows observed overlap in occupied environmental space between the modern and Maastrichtian.

| **Turtle family/ecotype** | **Niche similarity** | **Niche stability** | **Niche expansion** | **Niche unfilling** |
| --- | --- | --- | --- | --- |
| Trionychidae | p = 0.01 (similarity Maastrichtian to modern)  p = 0.01(similarity modern to Maastrichtian) | ~0.69 | ~0.31 | 0.03 |
| Chelydridae | p = 0.01 (similarity Maastrichtian to modern)  p = 0.02 (similarity modern to Maastrichtian) | ~0.72 | ~0.72 | 0 |
| Freshwater | p = 0.03 (similarity Maastrichtian to modern)  p = 0.06 (similarity modern to Maastrichtian) | ~0.90 | ~0.10 | ~0.01 |
| Terrestrial | p = 0.21 (similarity Maastrichtian to modern)  p = 0.16 (similarity modern to Maastrichtian) | ~0.23 | ~0.77 | ~0.12 |

**Table 5** Significance of environmental niche similarity tests for testudine families and ecotypes, and niche stability, expansion and unfilling of niche margins in environmental space between the Maastrichtian and modern.

| **Families in freshwater ecotype (modern)** | Carettochelyidae, Chelydridae, Chelidae, Dermatemydidae, Emdidae, Geoemydidae, Kinosternidae, Pelomedusidae, Trionychidae |
| --- | --- |
| **Families in freshwater ecotype (Maastrichtian)** | Adocidae, Baenidae, Bothremydidae, Chelydridae, Compsemydidae, Dermatemydidae, Dortokidae, Haichemydidae, Kinosternidae, Lindolmemydidae, Macrobaenidae, Pelomedusidae, Solemydidae, Trionychidae |
| **Families in terrestrial ecotype (modern)** | Testudinidae |
| **Families in terrestrial ecotype (Maastrichtian)** | Nanshiungchelyidae |

**Table 6** Composition of testudine ecotypes (freshwater and terrestrial) used for the modern and Maastrichtian niche analyses.

| **ENM calibration** | **Average test AUC** |
| --- | --- |
| Trionychidae US calibration | 0.64 |
| Trionychidae Asia calibration | 0.70 |
| Terrestrial ecotype US calibration | 0.71 |
| Terrestrial ecotype Asia calibration | 0.78 |
| Terrestrial ecotype Africa calibration | 0.57 |

**Table 7** Average test AUC scores for trionychidae and terrestrial ecotype continental modern ENM calibrations, projected to global modern environmental layers.

**Figures 1a – h** show MaxEnt jackknife estimates of variable importance to ENM fit. Each variable is excluded in turn and a model created with the remaining variables. A model is then created using each variable in isolation. This identifies of those variables that contribute most to model test gain, as well as those that contain most information not present in the other variables (Phillips et al. 2006).

**Figure 1a** Maxent jackknife tests of averaged variable importance for modern freshwater ecotype global ENMs.

**Figure 1b** Maxent jackknife tests of averaged variable importance for modern terrestrial ecotype global ENMs.

**Figure 1c**: Maxent jackknife tests of averaged variable importance for modern Trionychidae global ENMs.

**Figure 1d** Maxent jackknife tests of averaged variable importance for modern Chelydridae global ENMs.

**Figure 1e** Maxent jackknife tests of averaged variable importance for Maastrichtian freshwater ecotype global ENMs.

**Figure 1f** Maxent jackknife tests of averaged variable importance for Maastrichtian terrestrial ecotype global ENMs.

**Figure 1g** Maxent jackknife tests of averaged variable importance for Maastrichtian Trionychidae global ENMs.

**Figure 1h** Maxent jackknife tests of averaged variable importance for Maastrichtian Chelydridae global ENMs.

**Habitat suitability**

**Trionychidae**

**Trionychidae**

**(ii)**

**(i)**

**a**

**b**

**Chelydridae**

**Chelydridae**

**(i)**

**(ii)**

**Habitat suitability**

**Freshwater ecotype**

**(i)**

**Freshwater ecotype**

**(ii)**

**c**

**Habitat suitability**

**Habitat suitability**

**d**

**Terrestrial ecotype**

**Terrestrial ecotype**

**(i)**

**(ii)**

**Figure 2** Maastrichtian MaxEnt ENMs projected to modern climate layers (i): Trionychidae (a), Chelydridae (b), freshwater ecotype (c) and terrestrial ecotype (d). Modern testudine occurrences (●) overlay the maps of habitat suitability (ii). Geographically filtered occurrences (1 per climate grid cell) are: Trionychidae: 1838, Chelydridae: 1378, freshwater ecotype: 11671, terrestrial ecotype: 2935.

**Temperature of the coldest quarter**

**Precipitation of the wettest month**

**Precipitation of the driest month**

**None**

**Figure 3** Climate variables most influential to type 1 novelty (areas with at least one variable outside the univariate range of climate space) in non-analogue climate regions, comparing the modern day and the Maastrichtian.

**Type 1 novelty and similarity**

**Figure 4 Availability of non-analogue climate space between the Maastrichtian and modern.** Green = regions of similar climate variables (0 indicates maximum similarity), red = regions with at least one variable outside the univariate range (type 1 novelty). The more negative values the type 1 novelty, the less similar the climates are in these regions.

**None**

**Temperature of the coldest quarter**

**Figure 5** Climate variables most influential to type 1 novelty (areas with at least one variable outside the univariate range of climate space) in non-analogue climate regions, comparing the Maastrichtian and the modern day.

**Sensitivity analyses**

Species ENMs vs family-level ENMS

Trionychidae

**Habitat suitability**

**a**

**Trionychidae**

**b**

***Apalone ferox***

**Habitat suitability**

***Apalone mutica***

**c**

**Habitat suitability**

**Figure 6** Modern MaxEnt ENMs projected to US modern climate layers for (a) Trionychidae – sub-sampled family occurrence data, (b) *Apalone ferox* and (c) *Apalone mutica*; (b) and (c) represent members of Trionychidae. Family and species occurrences (●) overlay the maps of habitat suitability.

**Figure 7a**: Maxent jackknife tests of averaged variable importance for modern Trionychidae US ENMs.

**Figure 7b**: Maxent jackknife tests of averaged variable importance for modern *Apalone ferox* US ENMs.

**Figure 7c**: Maxent jackknife tests of averaged variable importance for modern *Apalone mutica* US ENMs.

**a**

**Habitat suitability**

**Trionychidae**

***Trionyx triunguis***

**b**

**Habitat suitability**

**Figure 8** Modern MaxEnt ENMs projected to African modern climate layers for (a) Trionychidae family occurrence data, (b) *Trionyx triunguis*. Family and species occurrences (●) overlay the maps of habitat suitability.

**Figure 9a**: Maxent jackknife tests of averaged variable importance for modern Trionychidae African ENMs.

**Figure 9b**: Maxent jackknife tests of averaged variable importance for modern *Trionyx triunguis* African ENMs.

Chelydridae

**a**

**Chelydridae**

**Habitat suitability**

**Habitat suitability**

***Macrochelys temminckii***

**b**

**c**

**Habitat suitability**

***Chelydra serpentina***

**Figure 10** Modern MaxEnt ENMs projected to US modern climate layers for (a) Chelydridae family occurrence data, (b) *Macrochelys temminckii,* (c) *Chelydra serpentina*. Family and species occurrences (●) overlay the maps of habitat suitability.

**Figure 11a** Maxent jackknife tests of averaged variable importance for modern Chelydridae US ENMs.

**Figure 11b** Maxent jackknife tests of averaged variable importance for modern *Macrochelys temminckii* US ENMs.

**Figure 11c** Maxent jackknife tests of averaged variable importance for modern *Chelydra serpentina* US ENMs.

Calibration extent

Trionychidae

**Trionychidae: US calibration**

**(i)**

**(ii)**

**a**

**Trionychidae: US calibration**

**Habitat suitability**

**Trionychidae: Asia calibration**

**b**

**Trionychidae: Asia calibration**

**(i)**

**(ii)**

**Habitat suitability**

**(ii)**

**Figure 12** Modern MaxEnt ENMs projected to modern global climate layers (i) for (a) Trionychidae (US calibration extent) and, (b) Trionychidae (Asia calibration extent)*.* Trionychidae occurrences (●) overlay the maps of habitat suitability (ii).

Terrestrial ecotype

**a**

**Habitat suitability**

**b**

**Terrestrial ecotype: Asia calibration**

**Terrestrial ecotype: US calibration**

**c**

**Habitat suitability**

**(i)**

**Terrestrial ecotype: US calibration**

**(ii)**

**(i)**

**Terrestrial ecotype: Asia calibration**

**(i)**

**Terrestrial ecotype: Africa calibration**

**Terrestrial ecotype: Africa calibration**

**(ii)**

**(ii)**

**Habitat suitability**

**Figure 13** Modern MaxEnt ENMs projected to modern global climate layers (i) for (a) terrestrial ecotype (US calibration extent), (b) terrestrial ecotype (Asia calibration extent), and (c) terrestrial ecotype (Africa calibration extent)*.* Terestrial ecotype occurrences (●) overlay the maps of habitat suitability (ii).

**a**

**b**

**c**

**d**

**Figure 14** Test for niche equivalency in occupied environmental space for: Trionychidae (a), Chelydridae (b), freshwater ecotype (c), terrestrial ecotype (d). Obeserved overlap (red bar) is lower than 95 % of simulated overlaps (grey bars), thus the assumption of niche equivalency is rejected for all four groups.

**Figure 15** Correlation circle showing the contribution of individual environmental variables to the two environmental gradient axes (environmental gradient 1 = 52.88 %, and environmental gradient 2 = 22.8 %). Environmental gradient 1 is mainly represented by precipitation of the wettest month and quarter and temperature seasonality. Environmental gradient 2 is represented by a combination of the remaining variables, including precipitation of the driest quarter, precipitation seasonality, mean temperature of the coldest quarter and mean temperature of the warmest quarter.

**References**

Barve, N., V. Barve, A. Jiménez-Valverde, A. Lira-Noriega, S. P. Maher, A. T. Peterson, J. Soberón, and F. Villalobos. 2011. The crucial role of the accessible area in ecological niche modeling and species distribution modeling*. Ecological Modelling* 222,1810–1819.

Elith, J., Kearney, M. & Phillips, S., 2010. The art of modelling range-shifting species. *Methods in Ecology and Evolution*, 1(4), 330–342.

Lunt, D. J., Farnsworth, A., Loptson, C., Foster, G. L., Markwick, P., O'Brien, C. L., Pancost, R. D., Robinson, S. A., and Wrobel, N., 2016, Palaeogeographic controls on climate and proxy interpretation. *Climates of the Past*, 12, 1181-1198.

Markwick, P. J. & Valdes, P. J., 2004. Palaeo-digital elevation models for use as boundary conditions in coupled ocean–atmosphere GCM experiments: a Maastrichtian (late Cretaceous) example. *Palaeogeography. Palaeoclimatology, Palaeoecology*, 213, 37–63.

Markwick, P. J., 2007. The palaeogeographic and palaeoclimatic significance of climate proxies for data-model comparisons, in *Deep time perspectives on climate change: marrying the signal from computer models and biological proxies*, edited by M. Williams, A.M. Haywood, J.F. Gregory and D.N. Schmidt. The Micropalaeontological Society Special Publications, Geological Society of London.

Phillips, S.J., Anderson, R.P. & Schapire, R.E., 2006. Maximum entropy modeling of species geographic distributions. *Ecological Modelling*, 190(3-4), 231–259.
